# Use of cannabinoid-based medicine among older residential care recipients diagnosed with dementia: study protocol for a double-blind randomised crossover trial

**DOI:** 10.1186/s13063-020-4085-x

**Published:** 2020-02-14

**Authors:** Amanda Timler, Caroline Bulsara, Max Bulsara, Alistair Vickery, Jill Smith, Jim Codde

**Affiliations:** 1 Institute for Health Research, University of Notre Dame Australia, Perth, WA Australia; 2 Emerald Clinics, Perth, WA Australia

**Keywords:** Dementia, Medicinal cannabis, Quality of life, Pain, Behavioural and neuropsychiatric symptoms of dementia (BPSD), Crossover trial

## Abstract

**Background:**

Dementia is a neurological condition that affects the cognitive and functional ability of the brain and is the leading cause of disability among those aged 65 years and above. More effective ways to manage dementia symptoms are needed because current treatment options (antidepressants and antipsychotics) can be ineffective and are associated with substantial side effects, including increased rate of mortality. Cannabinoid-based medicine (CBM) has shown an ability to inhibit some symptoms associated with dementia, and the adverse effects are often minimal; yet, little research has explored the use of CBM among this population.

**Aim:**

To monitor the safety of a purified dose of CBM oil (3:2 delta-9-tetrahydrocannabinol:cannabidiol) on behaviour symptoms, quality of life and discomfort caused by pain.

**Methods/design:**

We will carry out an 18-week, randomised, double-blind crossover trial that consists of a 2-week eligibility period, two 6-week treatment cycles, and two 2-week washout periods (between both cycles and after the second treatment cycle). We aim to recruit 50 participants with dementia who are living in residential aged-care facilities. The participants will be randomised into two groups and will receive a dose of either CBM oil or placebo for the first treatment cycle and the opposite medication for the second. Data will be collected using the Neuropsychiatric Inventory Questionnaire, the Cohen-Mansfield Agitation Inventory, the Quality of Life in Alzheimer’s Disease questionnaire, and the Abbey Pain Scale on seven occasions. These will be completed by the participants, aged-care staff, and nominated next of kin or family members. The participants’ heart rate and blood pressure will be monitored weekly, and their body composition and weight will be monitored fortnightly by a research nurse, to assess individual dose response and frailty. In addition, pre- and post-surveys will be administered to aged-care staff and family members to understand their perceptions of CBM and to inform proposed focus groups consisting of the aged-care staff and next of kin.

**Discussion:**

The study design has been informed by medical professionals and key stakeholders, including those working in the residential aged-care industry to ensure patient safety, collection of non-invasive measures, and methodological rigor and study feasibility.

**Trial registration:**

Australian New Zealand Clinical Trials Registry, ACTRN12619000474156. Registered on 21 March 2019

## Background

Dementia is a collection of symptoms that progressively reduces the cognitive and functional ability of the brain [1] and affects memory, intellect, rationality, social skills and physical functioning [2]. The symptoms associated with dementia present themselves in a variety of ways and can include depression, frustration, clinginess, forgetfulness, wandering, sexual aggression, hoarding, sleep disturbances and ‘the sundowner effect’ (increased manifestations of challenging behaviours at the end of the day [3]). Severe cognitive fluctuations in patients with dementia have been associated with an individual’s impaired ability to engage in activities of daily living, including social interactions and poorer quality of life (QOL) [4]. The slow progression and degeneration of dementia require that the affected individual receive additional support and assistance to remain at home or ultimately admission into residential aged-care facilities with 24-h care.

Dementia is the second leading burden of disability among Australians aged 65 years and older, and the burden of disease is expected to increase exponentially over the next 30 years [5]. Alzheimer’s disease is the most common cause of dementia and affects approximately 50–70% of the elderly with dementia. Pharmacological management of behavioural and physical symptoms of dementia is currently the most common treatment option, and many are prescribed medications such as off-label antipsychotics, sedative/hypnotics, anxiolytics, acetylcholinesterase inhibitors, and antidepressants to mask and alleviate the array of dementia symptoms [6, 7]. Medications such as aripiprazole, olanzapine, risperidone and memantine have been shown to reduce troublesome behaviours [8], although unclear guidelines are often provided for administration [2, 7]. This results in polypharmacy and its inherent risks, with numerous medications being prescribed for a longer duration than recommended [9]. Many of these medications lead to a number of substantial side effects [10], including increased rate of stroke and mortality [11].

Cannabinoid-based medicines (CBMs) have been shown to improve dementia symptoms such as aggression and agitation [12, 13], and they appear to be safer to prescribe than other pharmacotherapies [3] because the adverse effects are often minimal [14]. For example, Weier and Hall [15] found sedation to be the only adverse effect among patients with dementia prescribed either cannabinoids or pharmacotherapies. While periods of euphoria, somnolence and tiredness were observed among those prescribed dronabinol, a synthetically derived delta-9-tetrahydrocannabinol (THC) [16], there were only a small number of adverse events (6 of 98) related to the administration of the synthetic THC similar to those manifested by the placebo [14, 17]. However, well-designed, randomised, double-blind, placebo-controlled trials need to be completed to understand the most efficacious formulation, safety profile, drug–drug interactions and true effect to determine the place of CBM in dementia [18, 19], allowing greater generalisability of these outcomes [20].

A range of CBMs (synthetic compounds such as dronabinol or nabilone or pure cannabinols) are available; however, the combination of cannabidiol (CBD; the non-psychoactive compound) and THC (the psychoactive compound) [21] appear to be most effective, because both compounds improve psychomotor activity, mood, sleep–wake cycles and eating behaviours [14, 22, 23]. THC and CBD interact with the endogenous cannabinoid systems CB1 and CB2 receptors [19, 23, 24], producing symbiotic neuroprotective effects. For example, in pre-clinical trials, THC is found to be a partial CB1 antagonist and improves immune function [25], encourages amyloidogenesis [14, 22, 23, 26, 27], reduces neuropsychiatric symptoms, reduces pain sensation [28], stimulates appetite [21–23, 29], and inhibits acetylcholinesterase, similarly to cholinesterase inhibitors such as donepezil [30]. CBD is an inverse CB1 agonist [31] that promotes neurogenesis and vasodilation within the brain; increases neuronal plasticity and cerebral blood flow [32]; prevents cell destruction; and has anti-inflammatory (neuroinflammation and peripheral inflammation), analgesic, anticonvulsant and anxiolytic properties [25]. CBD is an important compound because it reverses the negative cognitive consequences and ameliorates the psychoactive properties of THC [21–23, 29].

Studies assessing the safety and efficiency of CBMs have shown many benefits among other neurodegenerative diseases, such as Parkinson’s disease [33, 34], epilepsy, post-traumatic stress disorder [18, 35], anxiety [12], and spasticity due to multiple sclerosis [36], with the use of CBM reducing benzodiazepine prescriptions by 45% [37]. However, only a handful of studies have investigated the use of CBM in patients with dementia [13, 14, 23]. Recently, an observational study monitored the use of a CBM medication over a 2-month period among ten females with severe dementia and found a 40% reduction in behavioural problems and 50% reduction in rigidity [38].

The pharmacodynamics and pharmacokinetics of THC (weeks 1–6, 0.75 mg; weeks 7–12, 1.5 mg) administered to ten participants with dementia was safe and well tolerated [14]. Administration of THC 2.5 mg in 11 patients with dementia demonstrated positive effects on mental state; dementia severity; and behavioural symptoms such as delusions, irritability, sleep and caregiver distress [23]. Studies reporting the use of dronabinol (2.5 mg daily) found improvements in anorexia and body weight, as well as less disturbed behaviours [16] such as agitation and motor behaviours, with no adverse effects observed [39]. Retrospective observations of dronabinol administration among 40 hospitalised patients indicated improvements in agitation and aggression, sleep duration, and meal consumption [13]. Two independent case studies monitoring the effects of nabilone (maximum dose 0.5 mg twice daily for 6 weeks) among elders with dementia found improvements in severe agitation and aggression [40], psychomotor activity, and smiling, as well as positive experiences among family members [41]. No changes in the number of falls or in balance (with eyes open) were reported among 18 participants administered 1.5 mg of oral THC twice daily [42], with recommendations suggesting higher doses (THC 1.5 mg three times daily), and longer study durations (great than 3 weeks) are needed to understand the true effects on behavioural symptoms, including QOL and activities of daily living [43]. Therefore, further research in this area is needed because many beneficial outcomes have been reported, although dosing, samples size, patient cohort (2–50 participants) and outcomes have varied in what are generally small studies with poor experimental designs.

### Aims

The primary aim of the present study will be to see if a purified CBM oil is safe and improves behavioural and neuropsychiatric symptoms of dementia (BPSD). In addition, two secondary aims of this study will include examining QOL and discomfort caused by pain among patients with dementia receiving CBM oil.

## Methods and general study design

This study has received approval from the Human Ethics Research Committee at the University of Notre Dame Australia. The study will use a parallel mixed methods design. The research methodology for this study is a phase II, randomised, placebo-controlled crossover trial. The design will include a 2-week eligibility (assessment) period, two 6-week-treatment cycles to allow each participant to take part in both the control and treatment cycles, and two 2-week washout periods (one between both treatment cycles and the other after the second treatment cycle). The 6-week treatment cycles have been selected on the basis of safety, pharmacodynamics, and pharmacokinetics as reported by Ahmed et al. [14], and a 2-week washout period has been shown to be safe and an appropriate length of time for cannabis to metabolise in older individuals [44].

In addition, residential aged-care staff and next of kin perceptions towards the use of CBM oil will be evaluated via surveys administered prior to and upon completion of the study. At the end of the second treatment cycle, the residential aged-care staff and family members will be asked to participate in a follow-up focus group to gather more in-depth information regarding individuals’ perceptions before and after the administration of CBM. Each participant will be in the trial for approximately 4 months (18 weeks), but the duration of the study will last over 12 months in order to recruit participants from a number of aged-care facilities.

### Participants and setting

Participants will be recruited through residential aged-care facilities. Residential aged-care facilities within Australia are government-funded organisations that provide additional support for families who may have a family member with dementia, whereby many move from their residential homes into a residential aged-care facility so that they can be monitored and provided with additional care. PASS^2019^ power analysis and sample size software (ncss.com/software/pass; NCSS, Kaysville, UT, USA) was used to derive the sample size. The Neuropsychiatric Inventory Questionnaire–Nursing Homes (NPI-NH) is the primary outcome measure. Total sample size for a 2 × 2 crossover design assuming a two-sided *t* test to detect a mean difference of 6 on the NPI-NH scale with a standard deviation of 13 (for the difference) is 40 for a power of 80% and significance level of 5%. Fifty participants will be recruited to allow for a 20% attrition rate. Participants will be eligible to participate if they live in a residential aged-care facility, are aged 65 years or older, have a diagnosis of mild dementia (indicated by a score ≥ 20 on the Mini Mental State Examination [MMSE]), are able to speak English, are known as compliant with taking medication, are not bedridden, and are able to provide informed consent. Participants will be excluded if they have certain health conditions, such as frontotemporal or Lewy body dementia; have other comorbidities, such as epilepsy, anorexia nervosa, comorbid psychiatric conditions, Parkinson’s disease, or congestive heart failure; have a history of myocardial infarction or anginal pain, stroke, liver disease, or renal disease; or are taking medications such as primidone, phenobarbital, carbamazepine, rifampicin, rifabutin, troglitazone, *Hypericum perforatum*, and valproic acid that may interact with cannabis metabolism.

The pre-/post-surveys and focus group discussions are an exploratory, qualitative component of this study, and thus a definitive sample size calculation cannot be determined at this stage. We estimate that six focus groups comprising six to eight participants, including two groups of residential care staff, activity staff (care staff who monitor daily activities and social engagement), and family members, will ensure that data saturation has been reached. Written informed consent will be obtained from all participants, including the residential aged-care staff and the next of kin.

### Rigor

Residential aged-care staff usually work within the aged-care setting for at least 3 months and therefore are likely to be working in the same facility for the duration of the 4-month trial. The same aged-care staff member will monitor the same participant(s) for the duration of the study and report any changes on the participants’ behalf. To be classified as a residential care staff member, the individual must spend at least two occasions per week with the participant. The same registered nurse will administer the medication for both treatment cycles.

### Recruitment

The residential aged-care clinical and general managers who have established relationships with the participants and their next of kin will promote the study to those they feel would be eligible to participate. This will be performed through face-to-face conversations.

### Randomisation

The randomisation process for this study will be done by creating a random number list using a 1:1 ratio allocation to ensure an equal number of cases in both the placebo group (*n* = 25) and the treatment group (*n* = 25) using Excel software (Microsoft Corp., Redmond, WA, USA). The determination of participant allocation will be completed by the laboratory manager in the drug manufacturing laboratory, with each case being provided a unique identification (ID) number (1–50). The primary researcher, who is responsible for recruitment, will provide the laboratory manager with the participant’s name to be sequentially matched against with the next available ID number. The laboratory will provide the pharmacy with both CBM and placebo in identical bottles labelled with the ID, but the medical practitioner and research team members will not know the order of treatment until the completion of the study. The pharmacist will place the participant’s name on the bottle before distributing the bottles to the aged-care facilities.

### Blinding

This is a double-blind study. Therefore, the laboratory manager will be the only individual who will know the group allocation for the participants. This is to ensure that the pharmacist, aged-care staff, medical practitioners, research nurse, family members/next of kin, participants and researchers are all blinded to the participant’s group allocation. Once the study is complete, the laboratory manger will un-blind the information by providing the primary researcher with a list of participants and their group allocation in order to conduct the analysis.

### Procedure

**Fig. 1:**
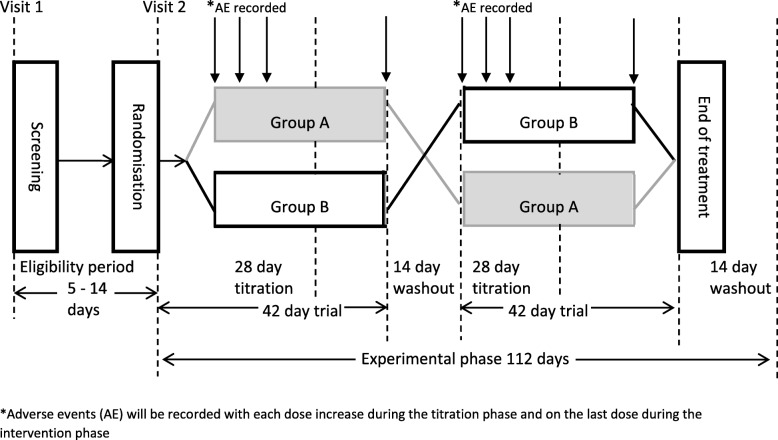
The key phases of the 18-week trial

The study will run for 18 weeks, comprising a 2-week eligibility period for screening and clinical assessment and a 16-week experimental phase encompassing two 6-week cycles of treatment and placebo separated by a 2-week washout period between the treatment cycles and a 2-week washout period following the completion of the second arm (Fig. 1).

#### Eligibility period

Individuals who express interest in participating in the study will initially be screened on the basis of inclusion criteria (described above). Following the initial screening process, potential participants will undergo a thorough clinical investigation by a geriatrician to ensure they have the cognitive capacity to provide informed consent using the MMSE. The MMSE [45] is the most widely used cognitive outcome measure to assess the severity of cognitive performance. It comprises 11 items, where a total score out of 30 can be calculated to assess the severity of dementia (25–30 = questionably significant, 20–25 = mild, 10–20 = moderate, 0–10 = severe). Those who seem suitable will be revisited by the geriatrician 1 week after the cognitive tests and will confirm that the participant has understood the purpose of the trial and has recalled the details of the study. Then the primary researcher will invite the eligible participants to enrol in the study and ask them to complete the consent form and provide some demographic and baseline information, including age, sex, education level, weight, medical history including comorbid illnesses, and prescribed medications. The participants will then be randomly allocated to treatment group A or B and receive either CBM oil or placebo for the first 6-week treatment cycle. No adjustments will be made to the participants’ currently prescribed medications.

#### Experimental phase

This phase of the study will take 16 weeks to complete. To minimise the risk of adverse events and variation in the maximum tolerated dose of CBM oil, each participant will receive one dose on the first and second days (2 pm) and two doses (9 am and 2 pm) for the reminder of both treatment cycles. A registered nurse will administer the dose along with a small meal (e.g., morning and afternoon tea), and the rate of titration will be monitored by the pharmacist to ensure it is appropriate for each individual. The participant will gradually receive an increased dose (titration) of the medication over several weeks, as shown in Table 1. During these weeks, the participant along with the care staff will record the presence of, and any change in, any potential adverse events that may be associated with the medication after the first dose, each afternoon when the dose is increased, and again on the final day of medication. If an adverse event is noted, the participant will revert to their previous best tolerated dose using the adverse events and safety protocol listed below.

**Table 1:** Titration administration, including dose and number of sprays

Day(s)	Week	Dose administered (daily)		Number of spray(s) per dose	Adverse events recorded (time)
		9 am	2 pm		
**1**, 2	1	–	**2.5 m**g	1	3 pm
**3**, 4	1	2.5	2.5	1	
**5**, 6	1	2.5	**5**	1–2	3 pm
**7**, 8	1	5	5	2	
**9**, 10	2	5	**10**	2–4	3 pm
**11**, 12	2	10	10	4	
**13**, 14	2	10	**15**	4–6	3 pm
**15**, 16	2/3	15	15	6	
**17**, 18	3	15	**20**	6–8	3 pm
**19**, 20	3	20	20	8	
**21**, 22	3/4	20	**25**	8–10	3 pm
**23**, 24	4	25	25	10	3 pm

Adverse events will be recorded 1 h after drug administration on days of dose increase (days bolded) and on the last day of the treatment period

An upper limit of 50 mg/day of THC will be permitted in those who do not experience any adverse events from the medication. Once a participant has reached their maximum tolerated dose (or a total of 50 mg/daily of THC), they will continue to receive that dose until the cessation of the 6-week period. The placebo group will follow a similar titration process using the indicated volumes shown in Table 1. They will continue to receive an increase in the volume of medication until they record an onset of an adverse event, at which time they will continue to take that volume of placebo until the end of the 6-week placebo cycle.

#### Management and administration of medication

The CBM oil (CogniCann; MGC Pharmaceuticals Ltd., Perth, Australia) will be provided in a sealed 10-ml glass spray bottle which contains a mix of THC and CBD in a 3:2 ratio (25 mg/ml THC; 17 mg/ml CBD) in a medium-chain triglycerides oil base. Each press of the vial will accurately dispense 100 μl of oil that contains 2.5 mg of THC. A total of 50 mg/day of THC and 34 mg/day of CBD can be administered for 4–5 days from one 10-ml glass spray bottle. CogniCann can be stored at room temperature (below 25 °C) for a total of 4 weeks. A certificate of analysis will be provided for each batch upon delivery.

The placebo will be administered in the same 10-ml glass spray bottle and collected following the procedures describe above. The placebo will comprise a terpene-based oil that contains esters that mimic the smell and taste of CBM.

The bottles of medication will be provided to the residential aged-care facilities by the affiliated pharmacist. The bottles will be delivered every week and collected again after 7 days of use (even if they are half-full) and returned to the pharmacy, where staff can determine how much was used (or left) and then dispose of the bottles to meet Therapeutic Goods Administration (TGA) requirements. At the start of the titration phase, one bottle will be administered for each participant (because the lower dose of 2.5 mg of THC allows for each bottle to hold 2–3 weeks of the medication). As participants begin to reach a higher dose (titration phase; *see* Table 1), two bottles will be provided on a weekly basis so that each participant will have sufficient medication to last for 7 days.

### Data collection

The aged-care staff, resident participants with dementia, and nominated next of kin will complete a total of four outcome measures on seven occasions throughout the study. The questionnaires take approximately 20 min to complete and will be completed three times during the first treatment arm (baseline [day 0], after maximum tolerated dose has been reached [day 24], and the end of the treatment cycle [day 42]), three times during the second treatment arm (baseline [day 56], after maximum tolerated dose has been reached [day 80], and the end of the treatment cycle [day 97]) and once following the 2-week washout period after the second treatment arm (day 112). The questionnaires will be administered by the primary researcher.

### Adverse events and safety protocol

An adverse events protocol will be put into place to minimise any potential harm or risks of receiving additional medication [14]. This will include participants reporting if they have experienced any adverse events 1 h after the increased dose has been administered (*see* Appendix). If moderate to severe adverse events are recorded (determined as ‘Somewhat worse’ (moderate) or ‘Much worse’ (severe) on the participant’s adverse event record) and these events have not ameliorated by the time for the next dose, the participant will receive the previous best tolerated dose. If the effects of the adverse event(s) have disappeared or become milder and do not interfere with the participant’s daily functioning or well-being, the registered nurse may increase the dose at the indicated rate. Recurrence of adverse events after two attempts to increase the dose will result in the participants remaining at their previous best tolerated dose for the remainder of the intervention period. A participant who experiences an adverse event will stay on the previous dose for another 2 days before the next dose is increased. At the beginning of the titration phase, a staff member at the aged-care facility will be vigilant in monitoring any acute adverse events such as dizziness, discoordination with a danger of falls and injury, and extreme fatigue. Any adverse events recorded will be reported to a facility line manager and will then be communicated to staff during shift changes.

Additional safety monitoring will be completed by a research nurse who will meet with each participant to discuss their adverse event records and measure their heart rate and blood pressure twice per week. In addition, the participant’s weight and non-invasive body composition measures such as lean body mass, bone mass, body fat percentage, and fat mass will be measured once per week using a portable scale. In addition, a nurse-led review will be completed 2 days into the washout periods to monitor the participant’s withdrawal symptoms once no more medication is being administered.

### Measures

**The Neuropsychiatric Inventory Questionnaire–Nursing Homes** (NPI-NH) [46] is a questionnaire that measures 12 neuropsychiatric symptoms (delusions, hallucinations, agitation, depression, anxiety, euphoria/elation, apathy/indifference, disinhibition, irritability, aberrant motor behaviour, night-time disturbances and appetite changes). The frequency and severity of each symptom is rated (4-point and 3-point Likert scales). A total score can be calculated by adding the first 10 domains together, and all 12 domain scores can be summed in special circumstances where neurovegetative symptoms are of interest, and a carer disruptiveness score (summing the disruptiveness score of the 10 [or 12] behavioural domains) can be calculated. The NPI-NH can be completed in approximately 10 min.

**The Cohen-Mansfield Agitation Inventory** (CMAI) is designed to assess agitation cross three domains, namely physically aggressive behaviour, physically non-aggressive behaviour and verbally agitated behaviour [47]. The CMAI comprises 29 items, uses a 7-point Likert scale (never = (1), less than once per week = (2), once or twice per week = (3), several times per week = (4), once or twice per day = (5), several times per day = (6), several times per hour = (7)), and measures four subscales: aggressive behaviour, physically non-aggressive behaviour, verbally agitated behaviour, and hiding and hoarding. A total score of 203 is calculated, with a higher score indicating a higher frequency in behavioural occurrence, and the measure takes approximately 5 min to complete.

**The Quality of Life in Alzheimer’s Disease** (QOL-AD) instrument is designed to measure aspects important for an individual’s QOL. The QOL-AD consists of 13 items using a 4-point Likert scale (poor = (1), fair = (2), good = (3), and excellent = (4)) and is designed for both self-report and proxy report [48]. The QOL-AD measures four domains (physical health, mental health, social, and function) and can be completed with people with a wide range of dementia severity [49]. A total score out of 52 is calculated, with a higher score indicating a higher QOL. The self-report version can be completed in about 10–15 min and the proxy report in about 5 min. A composite score can also be calculated (participant QOL-AD × 2 + carer QOL-AD × 3).

**The Abbey Pain Scale** comprises six items assessing vocalisation; facial expression; change in body language; and behavioural, physiological and physical changes [50]. This questionnaire uses a 4-point Likert scale (absent = 0, mild = (1), moderate = (2), severe = (3)), and a total score out of 18 is calculated. The severity of pain is indicated as mild (score of 3–7), moderate (8–13) and severe (14+) and can be completed in less than 5 min.

### Process evaluation outcomes

The one-page pre- and post-surveys will be administered to aged-care staff and next of kin at the beginning of the first treatment cycle and at the end of the second treatment cycle. These surveys comprise seven to nine questions regarding individuals’ perceptions towards CBM oil use and the symptoms of dementia they find most challenging. A total of six questions will be asked during the focus group discussions. These questions relate to positive and negative observations among those taking CBM oil, as well as changes in perceptions, knowledge and benefits regarding the use of CBM use.

### Data analysis

#### Quantitative

The results of questionnaires completed on behalf of the aged-care staff, participants and family members will be analysed using IBM SPSS Statistics version 25 software (IBM, Armonk, NY, USA). The responses from the aged-care staff will be the main responses considered for analysis. Where available, participants and family responses will be included for secondary analysis. To examine group differences, the participants will be categorised according to their treatment cycle group allocation (group A or group B). Descriptive statistics will be derived. Each variable will be tested for normality. For those variables that meet the normality assumption, two-sided paired and/or independent *t* tests will be used to examine group differences within and between groups. If the normality assumption is violated, then non-parametric tests such as the Wilcoxon signed-rank test will be used. Within-subject differences of the four measurements between the first and second washout periods will be tested using paired *t* tests to ensure that the washout phase is long enough to rule out any carryover effects [51, 52]. All data collection points will be examined using general linear mixed modelling techniques to see if any changes in behaviour, QOL or pain have occurred over the duration of both treatment cycles. The covariates of weight, average dose of medication, and baseline measures will be controlled for in each model, and any interactions will be tested and reported. The proportion of adverse events during the CBM and placebo phases will be tested and reported for each individual. NPI-NH is the primary outcome measure for this study. All other measures (CMAI, QOL-AD, and Abbey Pain Scale) have been included for secondary analysis. The CMAI will be analysed using the reliability change methodology compared with the NPI-NH to allow small changes to be reported [53]. The α-value will be set at 0.05. In the instance where a participant withdraws halfway through a treatment cycle, the information collected prior to withdrawal will be retained in the study because their personal information will have been de-identified. The data management of the information collected will follow standard university procedures, be stored in a locked cabinet for a period of 15 years, be stored on a password-protected computer, and be backed up regularly in a secure format.

#### Qualitative

NVivo 12 software (QSR International, Doncaster, Australia) will be used for qualitative data management and assistance in the analysis of both the pre- and post-surveys and in the focus groups. Qualitative content analysis will be used to analyse the surveys to assess similarities and differences between responses. The focus group results will be transcribed verbatim, and the transcripts will be thematically analysed by repeated readings and a subsequent open coding process followed by line-by-line coding to identify key themes. To avoid bias, a triangulated approach including reflectivity by the primary researcher during the interview process, member checking to establish confirmability, and verbatim quotes to establish credibility will be used. The primary researcher and the research team at the University of Notre Dame Australia will have access only to the final data set. The data will be stored in university computers on a locked storage drive.

## Discussion

To our knowledge, this is one of the first trials within Australia to evaluate the use of a purified CBM oil at the individual level among those with dementia to examine behavioural effects, QOL, and pain and discomfort. Only a handful of crossover trials have been conducted [14, 16, 41, 42, 54], although the majority have used fixed doses and have not incorporated an individually tailored dosing regimen. Soto et al. [28] reviewed 18 randomised clinical trials examining drugs prescribed for agitation and aggression and found large variations not only in the chosen questionnaires to measure these symptoms but also in the inclusion criteria. On the basis of their results, Soto et al. [28] suggested that trials lasting 9–12 weeks were adequate for assessing an acute response, whereas longer trials (6–12 months) were effective for assessing the stability of a response. The 18-week duration of this study is appropriate to assess the initial dose response [28], and the trial design is also reflective of other protocols of crossover studies and includes two 2-week washout periods to ensure patient safety and a chance for the medication to metabolise out of the body. For example, Babalonis et al. [31] designed a protocol to examine the use of a THC:CBD oromucosal spray among post-stroke spasticity patients and included two 4-week treatment cycles with a 2-week washout period between both cycles.

There are a number of strengths of our study. First, all participants will have a medical diagnosis of dementia. This ensures that the diagnosis is in line with the criteria of the *Diagnostic and Statistical Manual of Mental Disorders, Fifth Edition* [55]. Second, the residential aged-care staff spend a large amount of time with the participants, so they will be able to observe small changes, leading to accuracy in recording of the results. When possible, these results will also be compared with information from the participants and their next of kin to examine similarities and differences. Beattie et al. [56] published a protocol paper outlining a national project to collect multiple QOL perspectives from the care staff, family members and those living with dementia. Comparisons between care staff, family members and self-report QOL scores showed a linear relationship between reporters, with the residents often rating their QOL higher than the care staff [57, 58].

As an additional precautionary step, two separate questionnaires assessing behavioural symptoms have been included to ensure that the smallest effects of CBM oil are observed. A strict safety protocol monitoring of adverse events and nurse-led reviews during the washout periods will also be followed to ensure participant safety, which has been included due to the average age of the participants, additional medications currently prescribed, and the likelihood of having a comorbid condition. Weier and Hall [15] suggested that the therapeutic benefits of CBM are observed among patients with dementia when administered alongside adjacent therapy or medication. In addition, the dose of medication will be titrated to ensure that each participant receives their best tolerated dose and minimises the onset of any adverse events. This process of ‘start low and go slow’ is reflective of other studies as well as government documentation from Queensland Health [59, 60]. Educational training with the aged-care staff will be completed before the first participant is recruited to prevent unexpected issues arising during the trial and to ensure that they are familiar with the structure of the overall research protocol as well as the questionnaires.

This study has used a holistic approach to gain more in-depth information about BPSD and to capture staff and family members’ perceptions of CBM. The inclusion of the qualitative phase is important because little research has been completed to gain understanding of personal views of CBM use and the effects thereof in this setting. This approach allows the researchers to understand the perceived strengths and challenges in the use of cannabis within an institutional setting. Many symptoms associated with dementia, such as wandering, agitation, aggression, and psychotic behaviours, contribute to fatigue and burnout experienced by many caregivers [61]. Feast et al. [62] reviewed the relationship of BPSD and well-being of informal caregivers (child-adult or spousal caregivers) and found that the most distressing symptoms for caregivers were depressive behaviours, agitation and aggression, apathy (including irritability), aberrant motor behaviours, and delusions.

A number of challenges have been identified in the proposed study. It may be difficult to find those with a dementia diagnosis who have the cognitive capacity to give informed consent, because many experience a loss of short-term memory, intellectual reasoning, rationality, and social skills [1, 2]. This may lead to difficulty in recruiting participants into the trial and excluding those who do not have the capacity to give informed consent; yet, they are potentially the ones who exhibit a greater number of behavioural occurrences. Those who experience mild to moderate dementia still exhibit symptoms such as depression and anxiety and both verbal and physical agitation [2]. However, due to the limited legislation regarding including those who do not have the cognitive capacity to give informed consent in research projects, this limits who can be included in this study. The replication of this study design to include those with a moderate to severe diagnosis would warrant further generalisability of the results. Irreversible progression of cognitive impairment, the associated complications and comorbidities, and frailty of the participants may lead to participants dropping out of the study. To accommodate this, we have included a 20% increase in the sample size. It is also difficult to know if the questionnaires chosen for this study are suitable and sensitive enough to measure the changes attributed to the use of CBM oil, because no ‘gold standard’ exists to measure BPSD. Therefore, both the NPI-NH and the CMAI have been selected to account for the small effects. The pharmacodynamics of the medication will be monitored during the treatment cycles through the use of non-invasive body composition measures and monitoring of heart rate, blood pressure and weight. These measures will be collected by a research nurse external to the aged-care facilities to improve the feasibility of the study, to avoid additional workloads placed on the aged-care staff, and to ensure that each participant is receiving their best tolerated dose. In addition, only one residential staff member has been selected to complete the questionnaires, because they spend a great deal of time with the residents and can report symptoms easily. Independent reviews have not been selected for this study, because they are unfamiliar to the participants, which may lead to inaccuracy in recording of the results.

## Trial status

The trial has been registered with the Australian New Zealand Clinical Trials Registry. The registration number is ACTRN12619000474156, and the trial was registered on 21 March 2019. Recruitment is set to begin in Feburary 2020. The approximate date that the recruitment will be completed is 20 July 2020.

### Supplementary information

**Additional file 1.** Standard Protocol Items: Recommendations for Interventional Trials (SPIRIT) 2013 checklist.

## Data Availability

Not applicable.

## Appendix

### Adverse events associated with THC and CBD

Adverse events will most likely occur within 1 h of administration of the medication but will often resolve within 48 h of treatment. Participants will report their adverse events to make sure the titration is appropriate for them.

Compared with how you normally feel, have you experienced a change in any of the symptoms described below in the last hour? Tick the most appropriate box below.

**Table:**

	No	Yes (tick box below)				
		Better	Somewhat better	Same as usual	Somewhat worse	Much worse
Drowsiness/fatigue						
Dizziness						
Dry mouth						
Sore throat						
Anxiety						
Nausea						
Memory decline or attention deficits						
Joy or euphoria						
Blurred vision						
Headache						
Paranoia						
Depression						
Lack of coordination						
Diarrhoea						
Appetite						
Sleep disturbance						

To be completed during the titration phase and following the last dose in each treatment arm

## Abbreviations

BPSD: Behavioural and neuropsychiatric symptoms of dementia
CBD: Cannabidiol
CBM: Cannabinoid-based medicine
CMAI: Cohen-Mansfield Agitation Inventory
MMSE: Mini Mental State Examination
NPI-NH: Neuropsychiatric Inventory Questionnaire–Nursing Homes
QOL: Quality of life
QOL-AD: Quality of Life in Alzheimer’s disease
TGA: Therapeutic Goods Administration
THC: Delta-9-tetrahydrocannabinol
